# Routine Eye Screening by an Ophthalmologist Is Clinically Useful for HIV-1-Infected Patients with CD4 Count Less than 200 /μL

**DOI:** 10.1371/journal.pone.0136747

**Published:** 2015-09-16

**Authors:** Takeshi Nishijima, Shigeko Yashiro, Katsuji Teruya, Yoshimi Kikuchi, Naomichi Katai, Shinichi Oka, Hiroyuki Gatanaga

**Affiliations:** 1 AIDS Clinical Center, National Center for Global Health and Medicine, Tokyo, Japan; 2 Department of Ophthalmology, National Center for Global Health and Medicine, Tokyo, Japan; 3 Center for AIDS Research, Kumamoto University, Kumamoto, Japan; University Hospital San Giovanni Battista di Torino, ITALY

## Abstract

**Objective:**

To investigate whether routine eye screening by an ophthalmologist in patients with HIV-1 infection is clinically useful.

**Methods:**

A single-center, retrospective study in Tokyo, Japan. HIV-1-infected patients aged over 17 years who visited our clinic for the first time between January 2004 and December 2013 and underwent full ophthalmologic examination were enrolled. At our clinic, ophthalmologic examination, including dilated retinal examination by indirect ophthalmoscopy was routinely conducted by ophthalmologists on the first visit. The prevalence of ophthalmologic diseases and associated factors including the existence of ocular symptoms were analyzed.

**Results:**

Of the 1,515 study patients, cytomegalovirus retinitis (CMV-R) was diagnosed in 24 (2%) patients, HIV retinopathy (HIV-R) in 127 (8%), cataract in 31 (2%), ocular syphilis in 4 (0.3%), and uveitis with unknown cause in 8 (0.5%). Other ocular diseases were diagnosed in 14 patients. The CD4 count was <200 /μL in all CMV-R cases and 87% of HIV-R. The prevalence of any ocular diseases, CMV-R, and HIV-R in patients with CD4 <200 /μL were 22%, 3%, and 15%, respectively, whereas for those with CD4 ≥200 /μL were 5%, 0%, and 2%, respectively. No ocular symptoms were reported by 71% of CMV-R cases and 82% of patients with any ocular diseases.

**Conclusions:**

Routine ophthalmologic screening is recommended for HIV-1-infected patients with CD4 <200 /μL in resource-rich settings based on the high prevalence of ocular diseases within this CD4 count category and because most patients with ocular diseases, including those with CMV-R, were free of ocular symptoms.

## Introduction

Antiretroviral therapy (ART) has dramatically improved the prognosis of patients with HIV-1 infection [1]. However, many patients are still diagnosed of advanced HIV-1 infection, with concurrent opportunistic infections [2,3]. HIV-1-infected patients are prone to develop ocular opportunistic infections, such as cytomegalovirus (CMV) retinitis, cryptococcosis, toxoplasmosis, and tuberculosis [4–6]. HIV retinopathy can also occur in patients with HIV-1 infection [7]. Of these ocular opportunistic infections, CMV retinitis occurs among patients with severely advanced HIV-1 infection, and can result in total blindness and increased mortality even in the ART era [8].

To date, there is no consensus on the importance of routine screening of HIV-1-infected patients for ophthalmologic diseases with dilated retinal examination using indirect ophthalmoscopy. The revised 2013 American CDC guidelines for opportunistic infections did not state recommendations for routine ophthalmologic screening for such patients and only described the expert opinion that “some specialists recommend yearly funduscopic examinations performed by an ophthalmologist for patients with CD4 counts <50 /μl” [9]. The aims of the present study were; 1) to determine the prevalence of ocular diseases according to CD4 count and 2) to determine factors associated with CMV retinitis and other ocular diseases, with a special focus on the presence of ocular symptoms, in order to assess the clinical utility of routine ophthalmologic screening by an ophthalmologist for patients with HIV-1 infection in a resource-rich setting.

## Methods

### Study design, setting, and participants

We conducted a single-center retrospective study to investigate the usefulness of routine ophthalmologic screening for patients with HIV-1 infection at AIDS Clinical Center, National Center for Global Health and Medicine (NCGM), Tokyo. AIDS Clinical Center is one of the largest referral centers for HIV-1 infection in Japan with more than 3,800 registered patients [10], and considering that the total reported number of patients with HIV-1 infection is 24,454 by the end of 2013, this clinic treats approximately 15% of the HIV-1 infected patients in Japan [11]. At our clinic, ophthalmologic examination, including dilated retinal examination using indirect ophthalmoscopy, is routinely conducted during the first visit to the clinic by an ophthalmologist, and if it is not possible during the first visit, patients are referred to an ophthalmologists during the second/third visit [12]. The following criteria were applied for enrollment of patients in the study. Inclusion criteria: 1) HIV-1-infected patients aged over 17 years who visited our clinic for the first time between January 2004 and December 2013 and underwent full ophthalmologic examination within one year from the first visit. Exclusion criteria: 1) patients who were already diagnosed with ocular diseases at the time of referral to our clinic, because the aim of this study was to evaluate the usefulness of routine ophthalmologic screening. Furthermore, it is often difficult to confirm retinal photography which are required for the diagnosis of CMV retinitis according to the standard ACTG criteria [12,13].

The study protocol was approved by the Human Research Ethics Committee of National Center for Global Health and Medicine (G-001623-01). Informed consent was waived because this study solely used the data gained from clinical practice. Patient information was anonymized and de-identified prior to analysis. The study was conducted according to the principles expressed in the Declaration of Helsinki.

### Definitions of ophthalmologic diseases and measurements

The results of the first ophthalmologic examination conducted in each patient were extracted from the medical records, together with any or no ocular symptoms. CMV retinitis which fulfilled the standard ACTG criteria of “confirmed CMV retinitis” was included as CMV retinitis cases which required a diagnosis by an experienced ophthalmologist and documentation of CMV retinitis by retinal photography [13]. The diagnosis of HIV retinopathy was based on the findings of microaneurysms, telangiectasia, retinal hemorrhages, and cotton wool spots (CWS) [7]. The diagnosis of ocular syphilis was based on 1) ophthalmological examination documenting findings specific to ocular syphilis and 2) evidence of syphilis infection defined by a positive serum quantitative rapid plasma reagin (RPR) (titer ≥8) and positive *Treponema pallidum* hemagglutination assay [14,15]. When ruling out other ocular diseases was difficult, the response to syphilis treatment was also taken into account. At our hospital, if the diagnosis of ocular diseases could not be confirmed, ophthalmological examination was repeated within one to four weeks, and the diagnosis was confirmed by at least two ophthalmologists. Old lesions considered inactive were excluded. All ocular diseases were reviewed and confirmed by an experienced ophthalmologist (SY) and a HIV expert (TN). The basic characteristics [age, sex, ethnicity, the day (if not available, the month) of diagnosis of HIV-1 infection, history of AIDS, route of HIV-1 transmission, and treatment status of HIV-1 infection (either treatment-naïve or experienced)], CD4 count, HIV-1 viral load, quantitative RPR were also collected. For the latter three variables, the data closest to and preceding the day of the first ophthalmologic examination were used. Quantitative RPR was routinely measured on the day of the first visit and on discretion of treating physician at our clinic. The variable CMV end-organ diseases other than retinitis was also collected; their diagnosis of each disease was based on the standardized ACTG criteria and confirmed within 4 weeks of ophthalmological examination [12,13]. The variables systemic steroid use, anti-CMV treatment, and chemotherapy were also recorded. They were defined as steroid, anti-CMV treatment, and chemotherapy, which were administered either orally or intravenously within one month preceding the ophthalmological examination [12].

### Statistical analysis

All analyses were performed and all results were presented at the subject-level, rather than eye level. Baseline characteristics were described for the entire study patients, those with CMV retinitis, HIV retinopathy, cataract, and ocular syphilis. A univariate logistic regression model was constructed to estimate the association of each variable with CMV retinitis, HIV retinopathy, and cataract, respectively, and variables with p <0.05 in univariate analysis were incorporated into the multivariate model. For CMV retinitis, the variables anti-CMV treatment and history of AIDS were not added to the multivariate model because of multicollinearity with CMV diseases other than retinitis and CD4 count, respectively, and because of the small number of cases with CMV retinitis. For cataract, the variable systemic steroid use was added to the multivariate model because systemic steroid use is an established risk factor for cataract [16,17]. Sex was not added to any of the models because the study included only a small number of female patients. Statistical significance was defined as two-sided *p* values <0.05. We used odds ratios (ORs) with 95% confidence intervals (95% CIs). All statistical analyses were performed with The Statistical Package for Social Sciences ver. 21.0 (SPSS, Chicago, IL).

## Results

**Fig 1:**
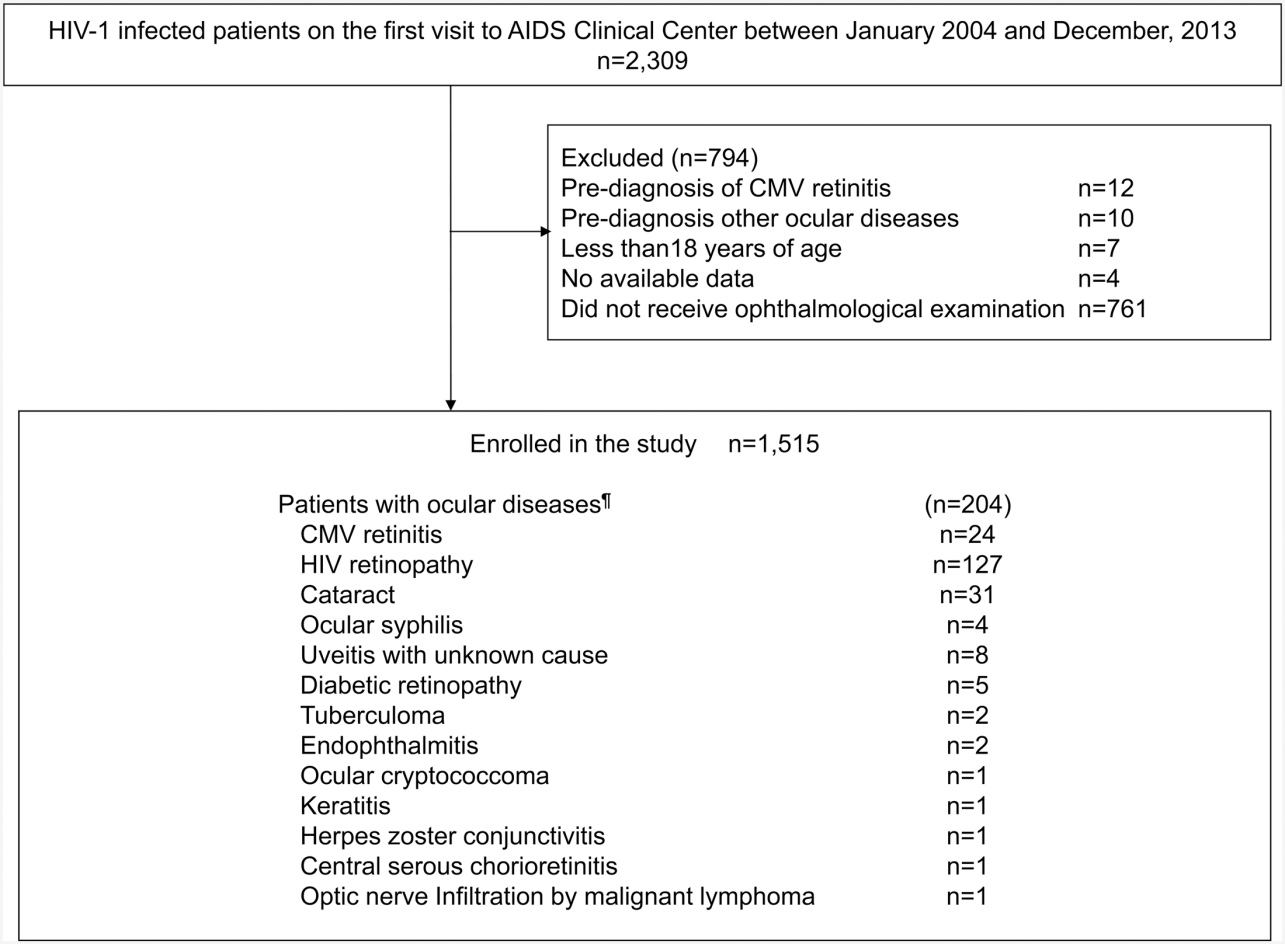
Patient enrollment process. ^¶^Two patients had both HIV retinopathy and cataract, one had both cataract and diabetic retinopathy, and one had both HIV retinopathy and diabetic retinopathy. CMV: cytomegalovirus.

1,515 (66%) patients were analyzed as the study patients (Fig 1). They were mostly Asian men who had sex with men and were treatment-naïve for HIV-1 infection (Table 1). The median CD4 count and HIV-1 load were 210 /μL [interquartile range (IQR) 66–353 /μL)] and 4.76 log_10_copies/mL (IQR 4.04–5.28 log_10_copies/mL), respectively. Median time from diagnosis of HIV-1 infection to ophthalmological examination was 1 month (IQR 0.3–2.6 months). Of the study patients, 204 (13%) presented with ocular diseases; ocular diseases included CMV retinitis in 24 (1.6%), HIV retinopathy in 127 (8.4%), cataract in 31 (2%), ocular syphilis in 4 (0.3%), uveitis with unknown cause in 8 (0.5%), and other diseases listed in Fig 1 in 14. Two patients had both HIV retinopathy and cataract, one had both cataract and diabetic retinopathy, and one had both HIV retinopathy and diabetic retinopathy.

**Table 1:** Baseline characteristics of study patients and those with various ocular diseases.

	Study patients n = 1515	Any ocular diseases n = 204	CMV retinitis n = 24	HIV retinopathy n = 127	Cataract n = 31	Ocular syphilis n = 4
Ocular symptoms, n (%)^¶^	50 (3)	27 (13)	7 (29)	10 (8)	10 (32)	2 (50)
Sex (male), n (%)	1428 (94)	199 (98)	24 (100)	123 (97)	31 (100)	4 (100)
Age^†^	36 (30–44)	42 (36–54)	42 (34–53)	40 (36–51)	63 (44–66)	37 (34–44)
Asian, n (%)	1456 (96)	200 (98)	22 (92)	124 (99)	30 (97)	4 (100)
CD4 count (/μl)^†^	210 (66–353)	67 (21–168)	27 (15–71)	51 (18–91)	224 (89–323)	181 (163–499)
HIV RNA load (log_10_/ml)^†^	4.76 (4.04–5.28)	5.22 (4.65–5.71)	5.28 (4.50–5.81)	5.36 (4.93–5.85)	4.59 (3.90–5.20)	4.35 (3.23–5.43)
Treatment-naive, n (%)	1320 (87)	185 (91)	21 (88)	118 (93)	26 (84)	4 (100)
CMV diseases other than retinitis, n (%)	17 (1)	8 (4)	2 (8)	6 (5)	0	0
Anti-CMV treatment, n (%)	36 (2)	11 (5)	3 (13)	7 (6)	0	0
Rapid plasma reagin titer ≥8, n (%)	210 (14)	27 (13)	4 (17)	14 (11)	3 (10)	4 (100)
Chemotherapy, n (%)	14 (1)	1 (0.1)	1 (4)	0	0	0
Systemic steroid use, n (%)	157 (10)	43 (21)	5 (21)	34 (27)	4 (13)	0
History of AIDS, n (%)	477 (32)	125 (61)	24 (100)	83 (65)	10 (32)	2 (50)
Route of transmission						
Homosexual contact, n (%)	1221 (81)	161 (79)	18 (75)	105 (83)	24 (77)	3 (75)
Heterosexual contact, n (%)	229 (15)	32 (16)	5 (21)	17 (14)	4 (13)	1 (25)
Contaminated blood product, n (%)	11 (1)	2 (1)	0	1 (1)	1 (3)	0
Injection drug, n (%)	24 (2)	3 (1.5)	0	1 (1)	1 (3)	0
Unknown, n (%)	30 (2)	6 (3)	1 (4)	3 (2)	1 (3)	0
Months between diagnosis of HIV-1 infection and ophthalmologic examination^†^ *	1 (0.3–2.6)	NA	NA	NA	NA	NA

^†^Median (interquartile range). CMV: cytomegalovirus, NA: not applicable.

Two patients had both HIV retinopathy and cataract.

^¶^Ocular symptoms were not assessed in five patients because of altered mental status

*Data for the day of diagnosis of HIV-1 infection are missing for 39 patients.

Table 2 shows the prevalence of ocular diseases according to CD4 cell count. Ocular diseases were identified in 81 (26%) of 308 patients with CD4 <50 /μL, 130 (27%) of 490 with CD4 <100 /μL, 162 (22%) of 731 with CD4 <200 /μL, and 28 (5.4%) of 784 with CD4 count ≥200 /μL. CMV retinitis was diagnosed in 24 (1.6%) of the 1,515 study patients, 14 (5%) of 308 patients with CD4 <50 /μL, 20 (4%) of 490 with CD4 <100 /μL, 24 (3%) of 731 with CD4 <200 /μL, and none among patients with CD4 count ≥200 /μL. For patients with CD4 count <200 /μL, three uveitis with unknown cause, two endophthalmitis, two diabetic retinopathy, two tuberculoma, one ocular cryptococcoma, and one keratitis were diagnosed other than those listed in Table 2.

**Table 2:** Prevalence of ocular diseases according to CD4 cell count.

	All patients n = 1515	CD4 <50 /μl n = 308	CD4 <100 /μl n = 490	CD4 <200 /μl n = 731	CD4 ≥200 n = 784
Any ocular diseases	204 (14)	81 (26)	130 (27)	162 (22)	42 (5.4)
CMV retinitis	24 (1.6)	14 (4.5)	20 (4.1)	24 (3.3)	0
HIV retinopathy	127 (8.4)	62 (20)	97 (20)	111 (15)	16 (2)
Cataract	31 (2)	2 (0.6)	9 (1.8)	15 (2.1)	16 (2)
Ocular syphilis	4 (0.3)	0	0	3 (0.4)	1 (0.1)

Data are numbers (percentages).

CMV: cytomegalovirus

At the time of ophthalmological examination, 50 patients (3%) of the total 1,515 patients complained of ocular symptoms (Table 1). Among 204 patients with any ocular diseases, only 27 (13%) had ocular symptoms. Similarly, only 7 (29%) out of 24 patients with CMV retinitis, 10 (8%) out of 127 with HIV retinopathy, 10 (32%) of 31 with cataract, and 2 (50%) of 4 with ocular syphilis, respectively, had ocular symptoms.

The median CD4 count of 24 cases with CMV retinitis was 27 /μL (IQR 15–71 /μL, range 7–158 /μL) (Table 1). Two (8%) patients also had CMV diseases other than retinitis, and three (13%) had received anti-CMV treatment. The results of multivariate analysis showed that the presence of ocular symptom versus no symptoms was significantly associated with CMV retinitis (OR 13, 95% CI 4.59–36.0, p<0.001) (Table 3). Lower CD4 count (per 100 /μL decrement, OR 3.9, 95% CI 1.85–8.30, p<0.001) was also significantly associated with CMV retinitis, whereas presence of CMV diseases other than retinitis showed a trend toward association that was not statistically significant (OR 3.9, 95% CI 0.78–19.1, p = 0.097).

**Table 3:** Uni- and multi-variate analyses to estimate the associations of various factors with cytomegalovirus retinitis.

	Crude model			Adjusted model		
	OR	95%CI	P value	Adjusted OR	95%CI	P value
Ocular symptoms versus no ocular symptoms	15	5.75–37.6	<0.001	13	4.59–36.0	<0.001
Age per 10 year increment	1.5	1.09–2.07	0.012	1.2	0.86–1.77	0.26
CD4 count per 100 /μl decrement	4.3	2.07–8.92	<0.001	3.9	1.85–8.30	<0.001
HIV-1 viral load per 1 log_10_copies/ml increment	1.5	0.96–2.28	0.079			
CMV diseases other than retinitis	8.9	1.93–41.5	0.005	3.9	0.78–19.1	0.097
Anti-CMV treatment	6.3	1.79–22.2	0.004			
History of AIDS	6.7	2.66–17.1	<0.001			
Antiretroviral therapy	1.0	0.29–3.27	0.96			
Chemotherapy	4.9	0.62–39.4	0.13			
Systemic steroid use	2.3	0.85–6.30	0.099			

Variables with p <0.05 in the univariate analysis were incorporated into the multivariate model. Anti-CMV treatment and history of AIDS were not added to the multivariate model because of multicollinearity with CMV diseases other than retinitis and CD4 count, respectively. History of AIDS was not added to the multivariate model because CMV retinitis is one of the AIDS-defining illnesses.

111 (87%) of 127 patients with HIV retinopathy had CD4 count less than 200 /μL (median 51 /μL, IQR 18–91 /μL, range 1–403 /μL). 34 (27%) patients were using systemic steroid, and 83 (65%) patients were with history of AIDS. In multivariate model, older age (per 10 year increment, OR 1.4, 95% CI 1.15–1.61, p<0.001), lower CD4 count (per 100 /μL decrement, OR 1.7, 95% CI 1.36–2.13, p<0.001), and higher HIV-1 load (per 1 log_10_copies/mL, OR 1.6, 95% CI 1.20–2.06, p = 0.001) were significantly associated with HIV retinopathy, whereas presence of ocular symptoms and history of AIDS showed a trend toward association that was not statistically significant (Table 4).

**Table 4:** Uni- and multi-variate analyses to estimate the associations of various factors with HIV retinopathy.

	Crude model			Adjusted model		
	OR	95%CI	P value	Adjusted OR	95%CI	P value
Ocular symptoms versus no ocular symptoms	3.0	1.44–6.06	0.003	2.2	0.99–4.76	0.052
Age per 10 year increment	1.5	1.31–1.78	<0.001	1.4	1.15–1.61	<0.001
CD4 count per 100 /μl decrement	2.3	1.92–2.84	<0.001	1.7	1.36–2.13	<0.001
HIV-1 viral load per 1 log_10_copies/ml increment	2.5	1.95–3.17	<0.001	1.6	1.20–2.06	0.001
History of AIDS	4.8	3.24–6.99	<0.001	1.6	0.97–2.57	0.064
Antiretroviral therapy	0.5	0.25–0.99	0.046	0.8	0.37–1.83	0.63
Systemic steroid use	3.8	2.44–5.81	<0.001	1.2	0.71–1.96	0.52

Variables with p <0.05 in univariate analysis were incorporated into the multivariate model.

31 patients with cataract were relatively old (median age 63, IQR 44–66), and had a median CD4 count of 224 /μL (IQR 89–323 /μL). In multivariate analysis, presence of ocular symptoms and older age were significantly associated with cataract (ocular symptoms, OR 13, 95% CI 4.86–33.4, p<0.001) (age per 10 year increment, OR 3.1, 95% CI 2.26–4.34, p<0.001), whereas systemic steroid use was not associated (OR 1.0, 95% CI 0.28–3.42, p = 0.97). In 4 patients with ocular syphilis, the titer of RPR was 256, 64, 64, and 16, and higher RPR value was associated with ocular syphilis (univariate analysis, per 2^n^ RPR titer increment, OR 1.6, 95% CI 1.18–2.15, p = 0.002, where RPR titer <8 was treated as RPR = 2).

## Discussion

In this single center cohort where an ophthalmologist routinely screens ocular diseases with dilated retinal examination using indirect ophthalmoscopy for HIV-1-infected patients who first visited the clinic [12], we aimed to determine whether routine ophthalmologic screening is clinically useful. The results of the present study showed the presence of ocular diseases and CMV retinitis in 26% and 5% of patients with CD4 <50 /μL, and in 22% and 3% of patients with CD4 <200 /μL, respectively, but only in 5.4% and 0% of patients with patients with CD4 count ≥200 /μL. Of the 24 patients with CMV retinitis, 14 patients had CD4 count <50 /μL, whereas 6 patients had CD4 50–99 /μL and 4 patients had CD4 100–199 /μL. Importantly, only 27 (13%) of 204 patients with any ocular diseases and 7 (29%) of 24 patients with CMV retinitis had ocular symptoms. The importance of early detection of ocular diseases, especially CMV retinitis is well established [18]. Based on the high prevalence of ocular diseases among patients with CD4 <200 /μL and high percentage of asymptomatic patients among those with ocular diseases, we recommend ophthalmological screening with dilated retinal examination using indirect ophthalmoscopy in HIV-1-infected patients with CD4 count of <200 /μL in resource-rich settings.

To our knowledge, this is the first study that implemented systemic screening by ophthalmologists for all HIV-1-infected patients during the first visit to the HIV referral clinic regardless of CD4 count. The study design allowed comparison of the prevalence of ocular diseases according to CD4 count. The result showed that the prevalence of ocular diseases among HIV-1-infected patients with CD4 ≥200 /μL was 5.4% (CMV retinitis 0%), far lower than 22% (CMV retinitis 3%) among patients with CD4 <200 /μL. Other studies that investigated the incidence and prevalence of ocular diseases among HIV-1-infected patients, such as the Longitudinal Study of the Ocular Complications of AIDS (LSOCA) [19], only enrolled AIDS patients with very low median nadir CD4 count of 30 /μL. Furthermore, in this study, most study patients underwent ophthalmological examination soon after the diagnosis of HIV-1 infection (median 1 month, IQR 0.3–2.6 months). Thus, the results of ophthalmological examination in this study would be similar to the results at the time of diagnosis of HIV-1 infection.

Little evidence is available for the clinical usefulness of routine ophthalmological screening in HIV-1-infected patients. Although both the 2013 American CDC guidelines for the treatment of opportunistic infections and 2013 Primary Care Guidelines by Infectious Diseases Society of America recommend referral of patients with CD4 count <50 /μL to an ophthalmologist for examination, the recommendation is mostly based on expert opinion [9,20]. It is well-known that CMV retinitis typically occurs in patients with CD4 count <50 /μL [21,22]. However, CMV retinitis can also occur in patients with CD4 >50 /μL [23], as in the present study the CD4 count was 50–199 /μL in 10 (42%) out of 24 CMV retinitis cases. Considering that 71% of CMV cases had no ocular symptoms, we recommend that routine screening by ophthalmologists of patients with CD4 <200 /μL is rational in resource-rich settings.

In the assessment of associated factors for each ocular disease, lower CD4 count and higher HIV-1 load were associated with HIV retinopathy, in agreement with the results of previous studies [24,25]. On the other hand, to our knowledge, this is the first study to report a significant association between old age and HIV retinopathy. HIV retinopathy is a well-known ocular disease characterized by micro aneurysms, telangiectasia, retinal hemorrhages, and CWS [7]. Pathogenesis and clinical significance of HIV retinopathy are not fully understood, and number of probable etiologies or associated factors, including viral immune complex, direct HIV infection of endothelial cells, and hyperviscosity due to red cell aggregation, have been suggested [7,26,27]. Clinically, most patients with HIV retinopathy do not have visual complaints [7] and CWS can disappear within a few weeks [28]. However, abrupt visual loss can occur in patients with HIV retinopathy [29], and HIV-induced CWS can cause permanent retinal destruction [26]. Our finding that old age is associated with HIV retinopathy might suggest the importance of active ophthalmologic screening for the elderly with HIV-1 infection.

The association between cataract and HIV-1 infection has been poorly documented. However, one Danish nationwide population-based cohort study reported almost twice higher risk of cataract surgery in patients with HIV-1 infection than in the general population [30]. One possible explanation for the susceptibility of HIV-1-infected patients to cataract is the occurrence of immune recovery uveitis/vitritis after the introduction of ART, with subsequent development of secondary ocular manifestations, including cataract [30–32]. The prevalence of cataract in this study was 2%, but it is difficult to directly compare this number with other studies, because older age is an established risk factor for cataract [33] and such comparison requires age matching. Although the Danish study identified that CD4 count ≤200 /μl and introduction of ART as risk factors for cataract [30], these findings were not reproduced in the present study.

Several limitations need to be acknowledged. First, due to the nature of a single-center cohort study, selection bias of study patients could not be ruled out. However, due to the very low prevalence of HIV-1 infection in Japan, our clinic treats approximately 15% of the HIV-1 infected patients in Japan [10,11]. Second, the majority of patients diagnosed with ocular diseases in the present study had HIV retinopathy, for which the clinical significance and sequelae are still not fully elucidated as described above. However, HIV-induced CWS can serve as a portal for CMV entry to the retina [34], and HIV retinopathy is an established risk factor for subsequent CMV retinitis [5,34,35]. Some experts even recommend ophthalmological examination every three months for patients with HIV retinopathy [7,27]. Third, 34% of the HIV-1-infected patients on the first visit to our clinic during the study period, including 12 patients with pre-diagnosed CMV retinitis, were excluded from the study (Fig 1), mostly because they did not receive ophthalmologic examination. If the characteristics of these patients differ substantially from the included cohort, this could influence the interpretation of the results.

In conclusion, the present study reported the results of systematic screening of HIV-1-infected patients by ophthalmologists with dilated retinal examination using indirect ophthalmoscopy among HIV-1-infected patients shortly after the diagnosis of HIV-1 infection. The prevalence of any ocular diseases and CMV retinitis were 22% and 3% in patients with CD4 <200 /μL, respectively, but were only 5.4% and 0% in patients with CD4 count ≥200 /μL. Furthermore, only 13% of patients with any ocular diseases and 29% of CMV cases complained of ocular symptoms. We recommend routine ophthalmologic screening by ophthalmologists for HIV-1-infected patients with CD4 count of <200 /μL in resource-rich settings.
